# Evolving Paralysis after Motor Vehicle Collision

**DOI:** 10.5811/cpcem2022.3.51179

**Published:** 2022-07-27

**Authors:** Nicole Prendergast, Youyou Duanmu

**Affiliations:** Stanford University School of Medicine, Department of Emergency Medicine, Palo Alto, California

**Keywords:** images, trauma, spinal epidural hematoma, operative neurological emergencies

## Abstract

**Case Presentation:**

An 85-year-old male who had been prescribed prasugrel presented to the emergency department (ED) after a motor vehicle collision and developed progressive neurological deficits. Computed tomography imaging demonstrated epidural thickening from the second through seventh cervical vertebrae, and magnetic resonance imaging was notable for a cervicothoracic epidural hematoma. The patient underwent emergent decompression with a favorable outcome.

**Discussion:**

Cases of traumatic spinal epidural hematomas are rarely seen in the ED. These are part of a small subset of operative neurological emergencies that benefit from urgent surgical intervention.

## CASE PRESENTATION

**Image 1:**
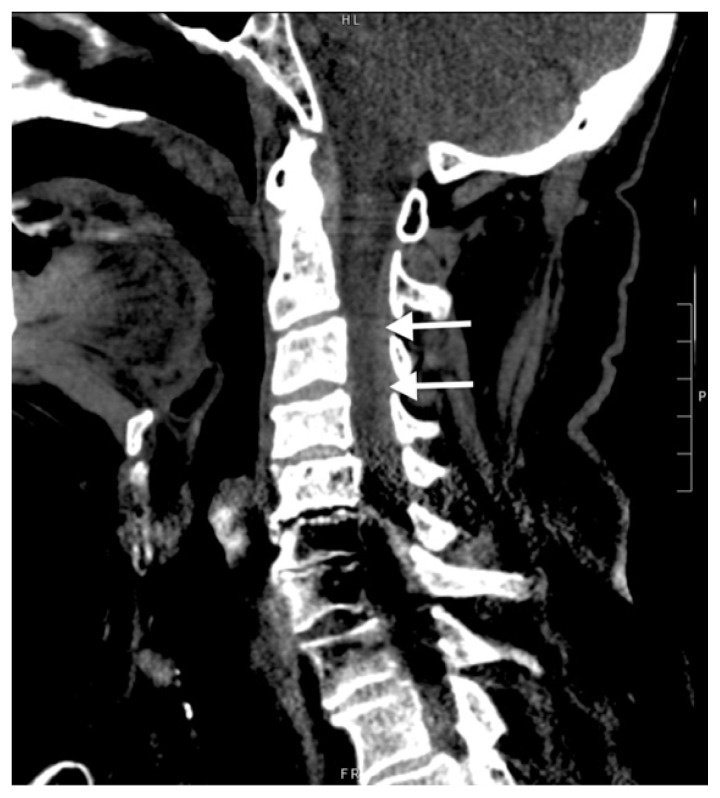
Sagittal non-contrast computed tomography of the cervical spine showing hyperdense dorsal epidural thickening (arrows) from second through seventh cervical vertebrae.

An 85-year-old male presented to the emergency department (ED) after a motor vehicle accident with abdominal pain, neck pain, and stool incontinence. The patient’s medication list included prasugrel, but it was unclear whether he was taking it. Exam was notable for cervical and thoracic spine tenderness, decreased rectal tone, decreased bilateral upper extremity sensation, and mild weakness of the right lower extremity. Cervical spine computed tomography (CT) demonstrated dorsal epidural thickening from the second through seventh cervical vertebrae (Image 1).

**Image 2:**
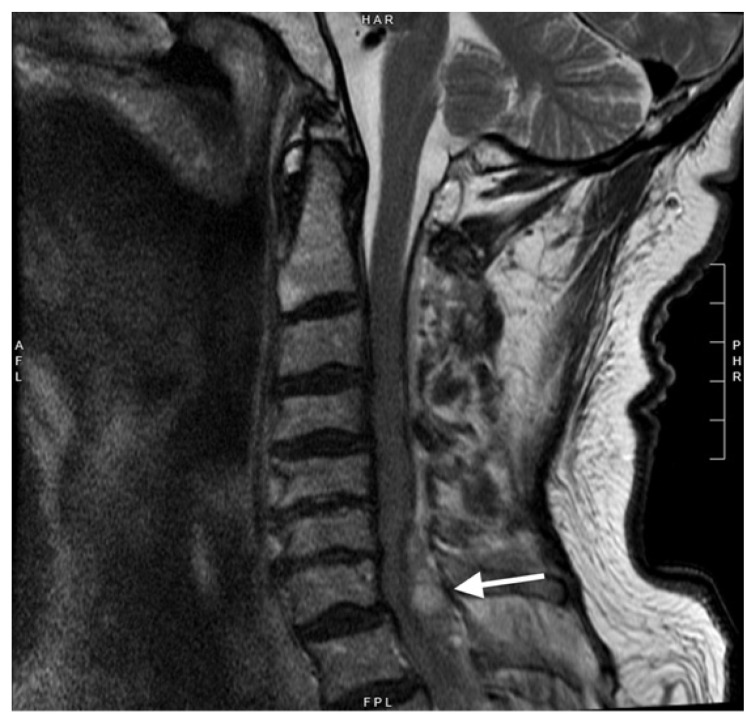
Sagittal T2-weighted, non-contrast magnetic resonance imaging of the cervical spine showing hyperintense dorsal epidural hematoma with cord compression most pronounced at the seventh cervical vertebra (arrow).

On reassessment, the patient had loss of sensation below the seventh thoracic dermatome and markedly diminished bilateral lower extremity strength and reflexes concerning for ascending paralysis due to spinal cord compression. Magnetic resonance imaging (MRI) of the complete spine showed a cervicothoracic epidural hematoma (Image 2).

Orthopedic spine surgery performed emergent decompression, and the patient experienced rapid postoperative improvement in strength and sensation.

## DISCUSSION

A spinal epidural hematoma is a collection of blood between the spinal canal dura and vertebrae.^1^ Spinal epidural hematomas can lead to permanent neurological deficit or death and, therefore, are a surgical emergency.^2^ Occurring in 1 per 1,000,000 annually, the cause is most commonly idiopathic (29.7%) followed by anticoagulation and vascular disorders.^2^ Those caused by trauma are rare (9.8%) and can be associated with minor injury.^1^,^2^ Symptoms involve radiating back or neck pain followed by neurological deficits consistent with evolving spinal cord compression including numbness, paresis, and loss of bowel or bladder function.^2^,^3^

Given the non-specific clinical findings, spinal epidural hematomas are challenging to diagnose.^1^ Non-contrast CT may show an epidural bleed as a hyperdense mass. An MRI with contrast (preferred if active extravasation or other spine pathology is suspected) or without contrast is the study of choice given the ability to estimate the location, size, and severity of compression.^2^ In the ED, medical management with dexamethasone and anticoagulant reversal, when indicated, can be initiated. 4 In patients with neurologic deficits, the definitive treatment is urgent surgical decompression, with operative intervention occurring under 12 hours of deficit onset associated with improved outcomes.^2^,^4^,^5^

**CPC-EM Capsule**

What do we already know about this clinical entity?

*A spinal epidural hematoma is a collection of blood between the spinal canal dura and vertebra that presents as back or neck pain with progressive neurological deficits*.

What is the major impact of the image(s)?

*Cases of traumatic spinal epidural hematomas are part of a small subset of neurological emergencies that benefit from early recognition and surgical intervention*.

How might this improve emergency medicine practice?

*Correlation of presentation with computed tomography and magnetic resonance imaging is essential to diagnose spinal epidural hematomas*.
